# Respiratory Syncytial Virus Induced Type I IFN Production by pDC Is Regulated by RSV-Infected Airway Epithelial Cells, RSV-Exposed Monocytes and Virus Specific Antibodies 

**DOI:** 10.1371/journal.pone.0081695

**Published:** 2013-11-26

**Authors:** Marcel A. Schijf, Michael V. Lukens, Debby Kruijsen, Nathalie O. P. van Uden, Johan Garssen, Frank E. J. Coenjaerts, Belinda van’t Land, Grada M. van Bleek

**Affiliations:** 1 Department of Pediatrics, The Wilhelmina Children’s Hospital, University Medical Center, Utrecht, The Netherlands; 2 Department of Immunology, Danone Research - Centre for Specialised Nutrition, Wageningen, The Netherlands; 3 Department of Medical Microbiology, University Medical Center, Utrecht, The Netherlands; 4 Utrecht Institute for Pharmaceutical Sciences (UIPS), Utrecht, The Netherlands; St. Jude Children's Research Hospital, United States of America

## Abstract

Innate immune responses elicited upon virus exposure are crucial for the effective eradication of viruses, the onset of adaptive immune responses and for establishing proper immune memory. Respiratory syncytial virus (RSV) is responsible for a high disease burden in neonates and immune compromised individuals, causing severe lower respiratory tract infections. During primary infections exuberant innate immune responses may contribute to disease severity. Furthermore, immune memory is often insufficient to protect during RSV re-exposure, which results in frequent symptomatic reinfections. Therefore, identifying the cell types and pattern recognition receptors (PRRs) involved in RSV-specific innate immune responses is necessary to understand incomplete immunity against RSV. We investigated the innate cellular response triggered upon infection of epithelial cells and peripheral blood mononuclear cells. We show that CD14^+^ myeloid cells and epithelial cells are the major source of IL-8 and inflammatory cytokines, IL-6 and TNF-α, when exposed to live RSV Three routes of RSV-induced IFN-α production can be distinguished that depend on the cross-talk of different cell types and the presence or absence of virus specific antibodies, whereby pDC are the ultimate source of IFN-α. RSV-specific antibodies facilitate direct TLR7 access into endosomal compartments, while in the absence of antibodies, infection of monocytes or epithelial cells is necessary to provide an early source of type I interferons, required to engage the IFN-α,β receptor (IFNAR)-mediated pathway of IFN-α production by pDC. However, at high pDC density infection with RSV causes IFN-α production without the need for a second party cell. Our study shows that cellular context and immune status are factors affecting innate immune responses to RSV. These issues should therefore be addressed during the process of vaccine development and other interventions for RSV disease.

## Introduction

The innate immune system is triggered upon recognition of pathogen associated molecular patterns (PAMPS) and sets the stage for the subsequent initiation of an appropriate immune response against an invading pathogen [1]. Toll like receptors (TLRs), cytoplasmic sensors (RIG-I like receptors RLRs, RIG-I, MDA5, LGP2) and nucleotide-binding oligomerization domain receptors (NOD-like receptors, NLRs) have unique specificities for pathogen-specific molecular structures [2]. In general pathogens contain several PAMPS, and in addition evasion mechanisms to suppress innate or adaptive immune responses. Combined with a specific entry locale in the body and the specific mode of interaction with host cell types, each pathogen induces unique tailored immune responses. RSV is a negative stranded RNA virus causing respiratory tract infections with sometimes a severe disease course especially in infants, immunocompromised and elderly individuals [3–5]. Due to high infection rates, RSV causes a high disease burden during yearly epidemics [6]. Important issues that need to be solved for RSV are the exact sequence of events and correlates of disease upon RSV infection during primary exposure and the reason for inadequate immune protection against reinfections that are frequent for this virus. 

 Viral infections are characteristically accompanied by type I interferon responses resulting from interaction of viral RNA with TLR7 and TLR3, for respectively single- stranded RNA or double-stranded RNA getting access to endosomal compartments [7]. In addition, cytoplasmic RNA helicase-like sensors such as RIG-I and MDA detect viral RNA upon infection when viral RNA replication intermediates are present in the cytoplasm [8–11]. Type I interferon induction is a crucial step to initiate the cellular antiviral response, but in addition affects the nature and efficacy of the induction of adaptive immune responses [12]. For RSV it has additionally been reported that the membrane Fusion (F) and attachment (G) glycoproteins interact with TLR2 (F) and TLR4 (both F and G) [13,14]. The importance of proper TLR interactions during the initiation of RSV specific adaptive immune responses have been revealed by a human vaccination trial and in animal models using a formalin-inactivated RSV vaccine. The lack of proper TLR signals provided by this and other inactivated RSV vaccines precluded high affinity antibody production [15]. Ineffective virus neutralization upon subsequent natural RSV exposure and strong Th2-biased T cell responses caused dramatic disease enhancement in vaccinated children and animals [15,16]. Current knowledge of innate immune responses induced by RSV comes from murine *in vivo* models [17–21], *in vitro* studies on the interaction of the virus with human cell lines [22–24], purified cells [25–29] or *in vitro* cultured dendritic cells [30–32]. In the present work, we studied the interaction of RSV with a mixture of peripheral blood mononuclear cells (PBMC) that represent different cell types, each with a specific set of pattern recognition receptors. We determined the innate response of individual cells in the mixture, the reciprocal effects of different innate immune responses by different cell subsets in the mixture and the role of virus specific antibodies in these responses. 

## Results

### Cell specific interaction and innate immune response to RSV

**Figure 1:**
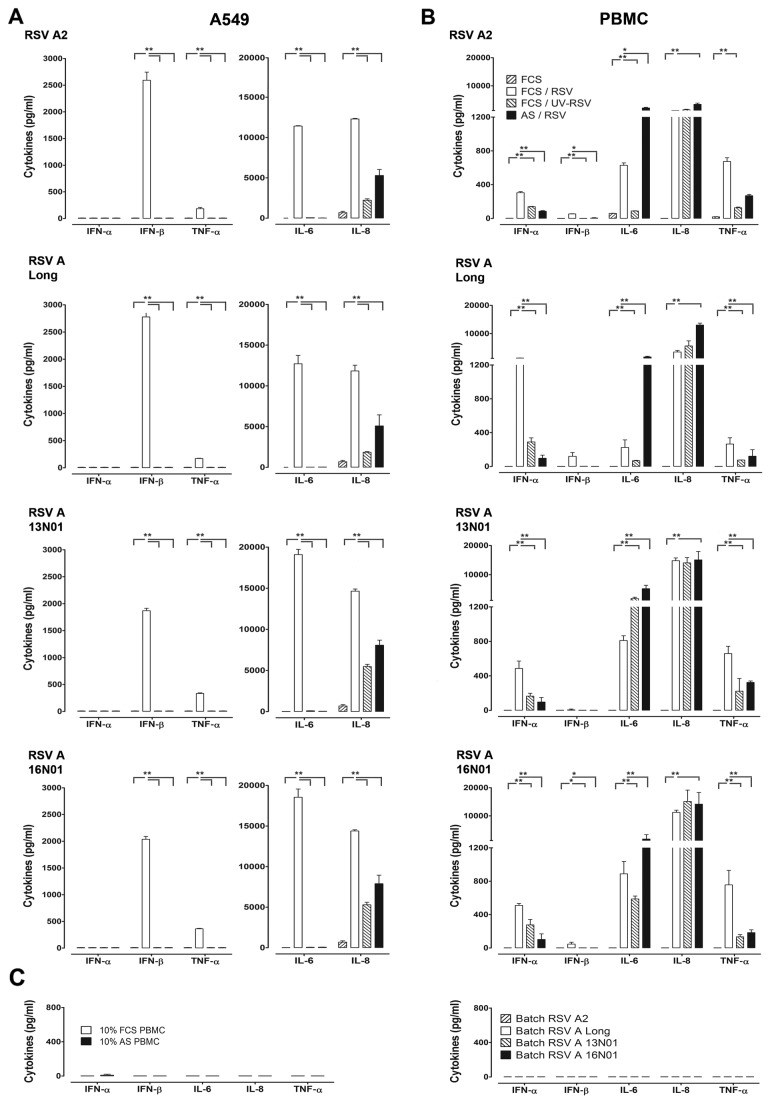
Cell specific innate immune response to RSV. Cytokine responses upon RSV exposure by lung epithelial cell line A549 (**A**) and human PBMC (**B**). Cells were co-cultured with the virus strains: RSV A2, RSV Long strain or 2 recent subtype A isolates, 13NO1 and 16NO1 at MOI 5. In addition to live RSV, the response to UV-inactivated and neutralized RSV in autologous serum (AS) was measured. After 20 hrs. incubation, cytokines in supernatant were measured by multiplex immunoassay and ELISA. In figure 1A the data represent mean values from duplicate experiments using human serum from four different donors. Figure 1B shows the cytokine responses in human PBMC exposed to RSV A2, representing the mean values measured in 6 different donors. For the other virus strains, means for four different donors are depicted whereby similar results were obtained in duplicate experiments. (**C**) Control measurements of cytokines produced in PBMC cultured in the presence of medium containing 10% FCS or autologous serum (AS, left) and cytokine measurements in the virus batches used in panels A and B (right). Data represent the mean ± SEM and were analyzed using two way ANOVA followed by a Bonferroni post-test, *P< 0.05, **P <0.01.

Host innate immune responses activated following RSV infection are suspected to contribute to RSV disease [33]. We studied the innate immune response induced by RSV in PBMC and an established cell line of human type II alveolar epithelial cells, A549. Each of the cells within PBMC and epithelial cells contain a cell specific characteristic set of innate immune receptors and potentially different attachment receptors. After exposure of PBMC and A549 cells to live, non-infectious UV-inactivated RSV or Ab-neutralized RSV for 20 hrs. at 37°C, we measured a set of cytokines in the supernatants of these cultures. 10% autologous serum of all donors, used in all experiments described in this study, completely prevented infection of A549 with all strains of RSV at a multiplicity of infection (MOI) of 5 (confirmed by the absence of surface staining by RSV specific monoclonal Ab to the RSV F protein on A549 cells exposed to RSV for 24 hours, data not shown). The complete inhibition of infection in the presence of 10% autologous serum or UV-irradiation was further confirmed by the complete absence of IFN-β and IL-6 production by RSV exposed A549 cells, which depends for epithelial cell lines on the access of viral RNA to intra-cellular RLR (Figure **1A**) [34,35]. The characteristic pattern of cytokines produced upon RSV exposure is shown for the A549 cell line and PBMC in Figure **1**. The antiviral type I IFN response and inflammatory cytokine response elicited by RSV A2 and RSV Long strain (a laboratory strain that lost the ability of natural strains to efficiently suppress type I interferon induction) and two recently isolated strains, 13N01 and 16N01, were compared at MOI 5. All four strains failed to induce IFN-α protein synthesis by A549 cells (Figure **1A**). The release of IFN-β protein by A549 cells depended on infection and was similar for all strains. In PBMC, all four strains induced significant amounts of IFN-α upon RSV infection and lower amounts when virus was inactivated by UV-irradiation or neutralized in autologous serum (Figure **1B**). RSV Long strain induced the highest amount of IFN-α presumably due to ineffective suppression of type I IFN production by NS proteins [35]. A549 epithelial cells mainly produced the inflammatory cytokines IL-6 and TNF-α in an infection dependent process, while IL-8 was induced by live RSV and still in significant amounts by non-infectious virus. However, while the IL-8 induction by infectious RSV was always present, induction of IL-8 by inactivated virus was variable between experiments and may depend on cell culture conditions [36]. The pattern and level of inflammatory cytokines produced in A549 cells were similar for all virus strains. The purified virus batches and supernatant of all PBMC cultures in autologous serum without virus were negative for the cytokines tested (Figure **1C**). 

RSV infection of PBMC induced the production of significant amounts of IL-6, IL-8 and TNF-α in addition to IFN-α, while IFN-β was barely detectible. Similar to A549 cells the amount of TNF-α produced by PBMC was suppressed when the virus was inactivated by UV irradiation or neutralised by autologous serum. In contrast IL-8 production was not affected by virus inactivation. The production of IL-6 was enhanced in the presence of autologous serum. At a lower viral dose (MOI 1), IL-6 production was only observed when PBMC were exposed to RSV in the presence of autologous serum (Figure **S1**). In summary, these results show that in A549 epithelial cells cytokine responses against RSV are most efficiently induced upon infection. PBMC respond to infectious virus with the production of IFN-α and TNF-α, while IL-6 is produced at higher amounts in the presence of human serum at a concentration whereby RSV is neutralized. These results therefore suggest different mechanisms of virus-cell interaction that contribute to the production of these inflammatory mediators.

### Binding efficiency of RSV is cell type specific and does not correlate with the susceptibility of the cells to infection

**Figure 2:**
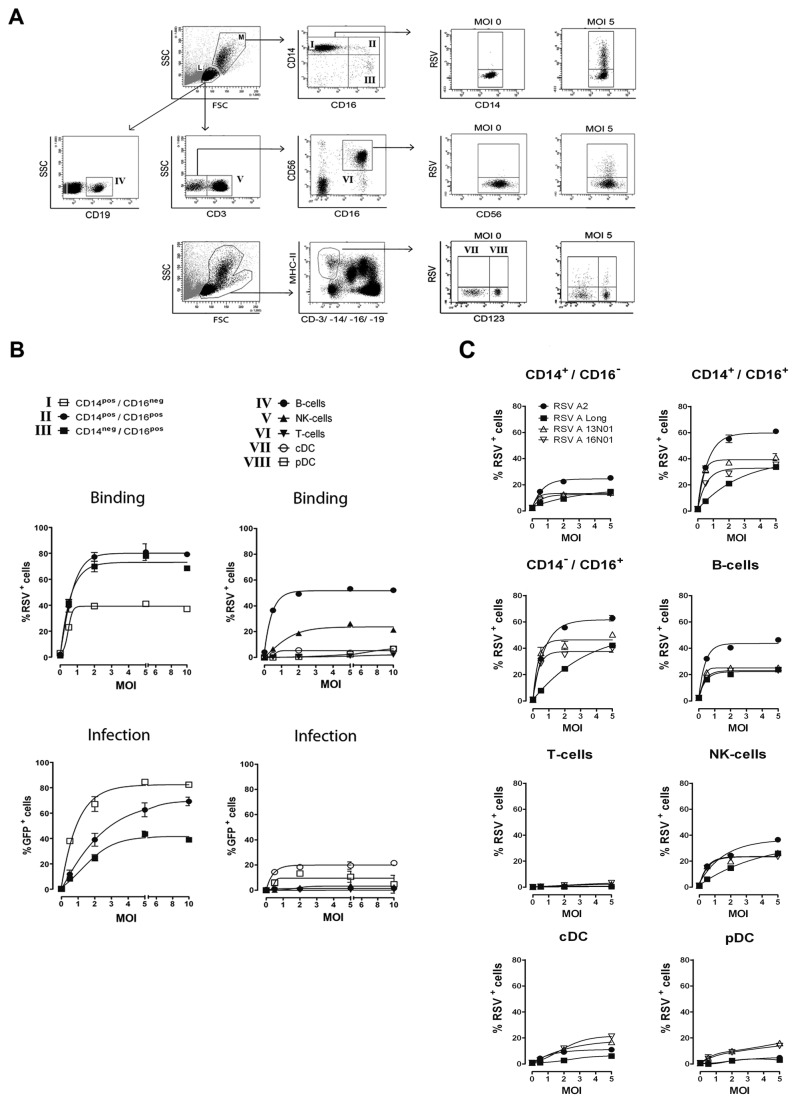
Binding efficiency of RSV is cell type specific and does not correlate with the susceptibility of the cells to infection. Binding and infection of rgRSV224 to different cell types in human PBMC was investigated. (**A**) FACS gating strategy for CD14^+^/CD16^neg.^, CD14^+^/CD16^+^ and CD14^neg^./CD16^+^ monocytes (in M: monocyte gate), CD19^+^ B-cells, CD3^+^ T-cells and CD3^neg^.CD16^+^CD56^+^ NK cells (in L: Lymphocyte gate). Dendritic cell populations were identified by CD3, CD14, CD16, CD19^neg^. (Lin^neg.^), MHC-II^high^, and either CD123^–^ (cDC) or CD123^+^ (pDC) (in the total live cell gate, M+L). (**B**) Binding of RSV to specific cell types was measured after 1 hour incubation at 4°C with polyclonal antibodies against RSV (upper figures). RSV infection was determined after 24 hrs. incubation at 37°C by the percentage of GFP expressing cells within a specific cell population (lower figures). (**C**) Cell type specific binding characteristics of 4 RSV A strains. Binding of RSV A2, RSV A Long, and clinical RSV A isolates 13N01 and 16N01 to specific cell types in PBMC was visualized after 1 hour incubation at 4°C. Binding was detected using polyclonal antibodies against RSV. Data represent the percentage of RSV positive cells within a population and are expressed as mean ± SEM of triplicate measurements within 1 donor. Experiments were performed in 5 different donors with similar results.

**Figure 3:**
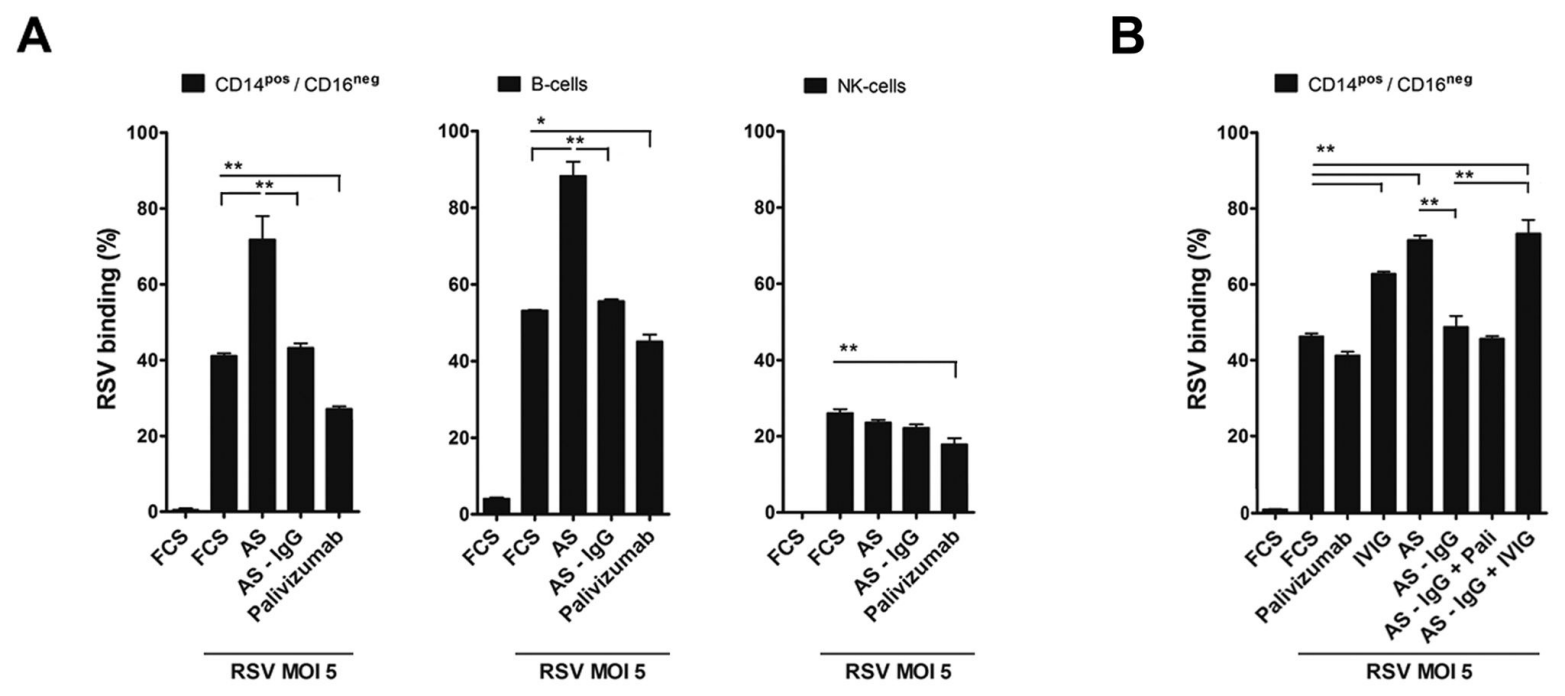
Virus specific polyclonal antibodies in human serum increase RSV binding to monocytes and B cells. (**A**) The effect of autologous serum, IgG-depleted serum and palivizumab in FCS on binding of RSV to monocytes, B cells and NK cells was measured after 1 hour incubation at 4°C. (**B**) Polyclonal antibodies (IVIG) increase binding of RSV to CD14^+/^CD16^neg^. cells. Data shown represent the mean ± SEM of triplicate measurements in two different donors (for A and B) and were analyzed using the Kruskal-Wallis test followed by Dunn's Multiple Comparison analysis, *P< 0.05, **P <0.01. Experiments were performed in 3 additional donors with similar results.

The different cellular cytokine profiles evoked upon interaction with live, dead and Ab-bound RSV suggests that the innate immune response depends on the interaction of the virus with pattern recognition receptors expressed on both the cell surface and in the cytoplasm. Thus, the capacity of the virus to bind to and/or penetrate the host cell determines the immune response initiated. To further investigate the cell specific response against RSV, we studied the interaction of RSV with the individual cell types in PBMC. We performed binding and infection studies with different multiplicities of infection (MOI) of recombinant green fluorescent protein (GFP)-expressing RSV A2 (rgRSV224) [37]. For RSV binding experiments we co-cultured PBMC with rgRSV224 for 1 hour at 4°C. Figure **2A** shows the gating strategy used to detect cell type specific binding and infection. Cell subpopulations in the monocyte gate (based on forward/sideward scatter) were classified as classical CD14^+^/CD16^neg.^ monocytes, CD14^+^/CD16^dim^ monocytes and CD14^neg^./CD16^+^ cells. We further identified CD123^+^ pDC and CD123^neg.^ MDC within the lineage-negative, HLA class II positive cell population in the total live cell gate. In the lymphogate we identified CD3^neg^./CD56^+^ (both CD16^+^ and CD16^neg.^) NK cells and CD3^+^ T cells. CD3^+^ cell subsets were not further divided into γ,δ T cells, CD4^+^ T cells, CD8^+^ T cells or NKT subsets because binding and infection characteristics were similar and extremely low in all subsets (data not shown). Figure **2B** (upper figures) shows representative graphs of the binding characteristics of rgRSV224 to the different cell subsets and identifies B cells in the lymphocyte gate and CD16^+^ cells in the monocyte gate as the cells that most efficiently bind RSV (respectively 52% and 79%). CD3^+^ T cells do not efficiently bind to RSV (1.9 %) and they are neither susceptible to infection. Classical CD14^+^/CD16^neg.^ monocytes and NK cells show intermediate binding to RSV (39% and 26% positive cells within the population respectively). Despite a quite significant binding of RSV to B and NK cells this results in insignificant infection within 24 hrs (Figure **2B**, lower right figure). In contrast binding efficiency to DC subtypes was low varying from 2-6% in different donors and with different virus preparations. However, infection was noted in 15-21% of these populations. Optimal binding to all cell populations was found at MOI 5 and optimal infection rates were reached at MOI 5 and up. However, at MOI 10 and 20 increased cell death was observed. The experiment depicted in Figure **2B** shows the data for binding studies with rgRSV224, the recombinant GFP-RSV strain. Similar results were obtained with RSV A2, RSV Long strain and the two natural isolates in binding experiments with PBMC from a different donor (Figure **2C**). Using different virus strains and virus batches the percentage of binding and also the kinetics of binding varied somewhat, but in all experiments we found the same hierarchy in the cell types that were binding the virus most efficiently, as well as a similar hierarchy in the susceptibility to infection. After 24 hours, about 84% of the CD14^+^/CD16^neg.^ monocyte sub-population, 62% of the CD14^+^/CD16^dim^ and 43% CD14^neg^./CD16^+^ monocytes were infected with rgRSV224 (Figure **2B**, lower left figure). These infection efficiency numbers show opposite hierarchy compared to the binding characteristics of these monocyte subpopulations. To determine the role of antibodies against RSV present in human serum on RSV binding capacity, we investigated the 3 cell populations (monocytes, B cells and NK cells) capable of binding RSV with high efficiency. Figure **3A** shows that autologous serum (AS) significantly enhanced binding of RSV. This was limited to monocytes and B cells. Binding of RSV to NK cells in AS remained unaffected. Depletion of the IgG fraction from autologous serum with protein G Sepharose beads, and reconstitution with polyclonal IgGs (IVIG^®^) showed that IgGs present in AS enhanced binding of RSV to monocytes (Figure **3B**). Only polyclonal antibodies and not the RSV-F specific monoclonal Ab palivizumab increased virus binding. In summary, RSV binds to various cell subtypes within the PBMC pool, but the level of binding does not correlate with the level of infection. DC and monocytes are the major cell types susceptible to RSV infection. 

### CD14^+^ monocytes are necessary for the inflammatory cytokine response against RSV in PBMC cultures

**Figure 4:**
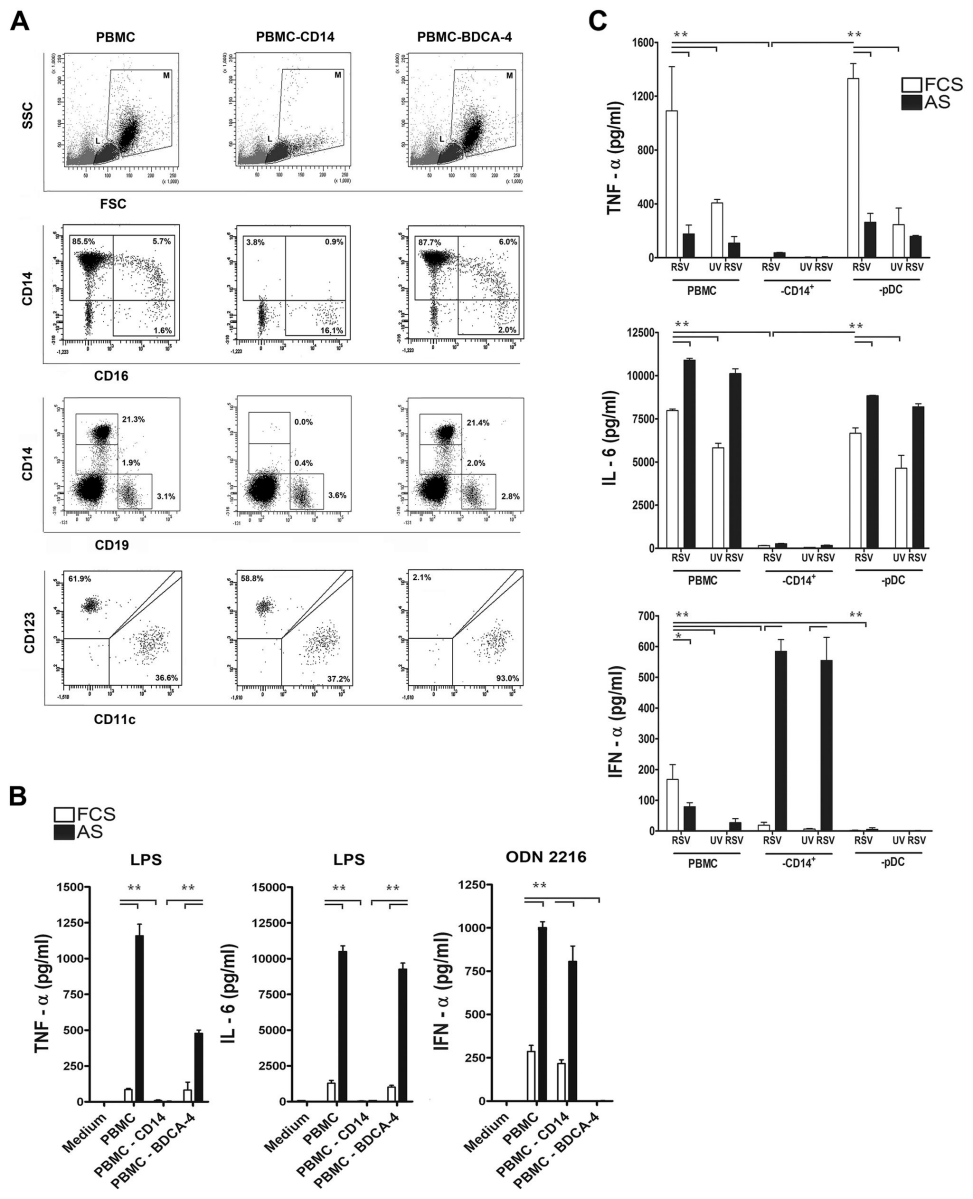
CD14^+^ cells are needed for the inflammatory cytokine response against RSV in PBMC cultures. (**A**) Confirmation of specific depletion of CD14^+^ and BDCA-4^+^ cells by FACS. M: monocyte gate, L: lymphocyte gate. Depletion of CD14^+^ cells leaves B cells (in M+L, CD19^+^ third row) and CD16^+^ monocyte/DC (in M, CD14^neg.^, CD16^+^, 2^nd^ row), cDC (in M+L, lineage^neg.^, MHCII^+^, CD11c^+^, 4^rd^ row) and pDC (in M+L, lineage^neg.^, MHCII^+^, BDCA-4^+^, 4^rd^ row) compartments intact. Depletion of BDCA-4^+^ cells removes pDC but does not affect CD14^+^CD16^neg.^, CD14^+^CD16^+^, CD14^neg^.CD16^+^ cells in the M gate (2^nd^ row), nor B cells (3^rd^ row) and cDC (4^rd^ row). The contribution of single cell types to anti-RSV cytokine responses in PBMC cultures was evaluated by depletion of specific cell populations. Cytokines in supernatant from the remaining cell populations were measured after 20 hrs. exposure to RSV or UV-RSV in the presence or absence of autologous serum. (**B**) Depletion of CD14^+^ cells and BDCA-4^+^ cells was confirmed by stimulation of depleted cell populations by TLR ligands, ultra-pure LPS to confirm the absence of TLR4^+^ (monocytes) and ODN 2216 to confirm the absence of TLR9^+^ (pDC). (**C**) PBMC, CD14 monocyte-depleted PBMC, or pDC-depleted PBMC were cultured with live and UV inactivated RSV (A2, MOI 5) in the presence or absence of 10% autologous serum. Data represent the mean ± SEM of 3 measurements in 1 donor and were analyzed using one way ANOVA followed by a Bonferroni post-test, *P< 0.05, **P <0.01. Experiments were performed in 3 different donors with similar results.

We next investigated the contribution of individual cell types in PBMC mixtures to the cytokine response upon RSV exposure, by removing single cell subsets. Removal of monocytes (CD14^+^ cells) or pDC (BDCA-4^+^ cells) was accomplished via magnetic cell separation. Specificity of depletion and composition of the remaining cell populations was checked by flow cytometric analysis and resulted in >98% depletion of CD14^+^ and BDCA-4^+^ cells, leaving the remaining cell populations intact (Figure **4A**). Cell specific depletion was also confirmed by the complete lack of IL-6, IL-10 and TNF-α production upon stimulation of CD14^+^ cell-depleted PBMC with LPS, a specific ligand for the CD14-TLR4 complex (Figure **4B**). Lack of IFN-α production upon stimulation with TLR9 ligand ODN 2216 confirmed pDC depletion. LPS and ODN 2216 stimulation in the presence of autologous serum resulted in increased cytokine production in PBMC. 

PBMC exposed to live and UV-inactivated RSV A2 produced cytokines IL-6 and TNF-α. This response depended entirely on the presence of CD14^+^ cells in the mixture while depletion of pDC did not affect these responses (Figure **4C**). Autologous serum increased IL-6 production by PBMC exposed to RSV, but suppressed virus induced TNF-α (Figure **4C**). The IFN-α response observed in PBMC cultures exposed to live RSV decreased after UV irradiation and depletion of either CD14^+^ cells or pDC. Moreover, the amount of IFN-α and TNF-α produced in response to live RSV varied between experiments and between virus batches (Figure **1B** and **4C**). IFN-α production was facilitated in CD14^+^ cell depleted PBMC in the presence of autologous serum (Figure **4C**). These results show that, although RSV binds to various cell subtypes within the PBMC pool (Figure **2**), CD14^+^ monocytes play a central role in the inflammatory cytokine response against RSV, but pDC are required for the production of IFN-α. pDC appear to be the exclusive source of IFN-α when RSV is presented in the presence of autologous serum to CD14^+^ cell depleted PBMC. However, this pathway of IFN-α production in the presence of serum is inhibited by CD14^+^ monocytes.

### RSV-specific antibodies inhibit IFN-α production in RSV-infected PBMC but enhance IFN-α production in CD14^+^ cell-depleted PBMC

**Figure 5:**
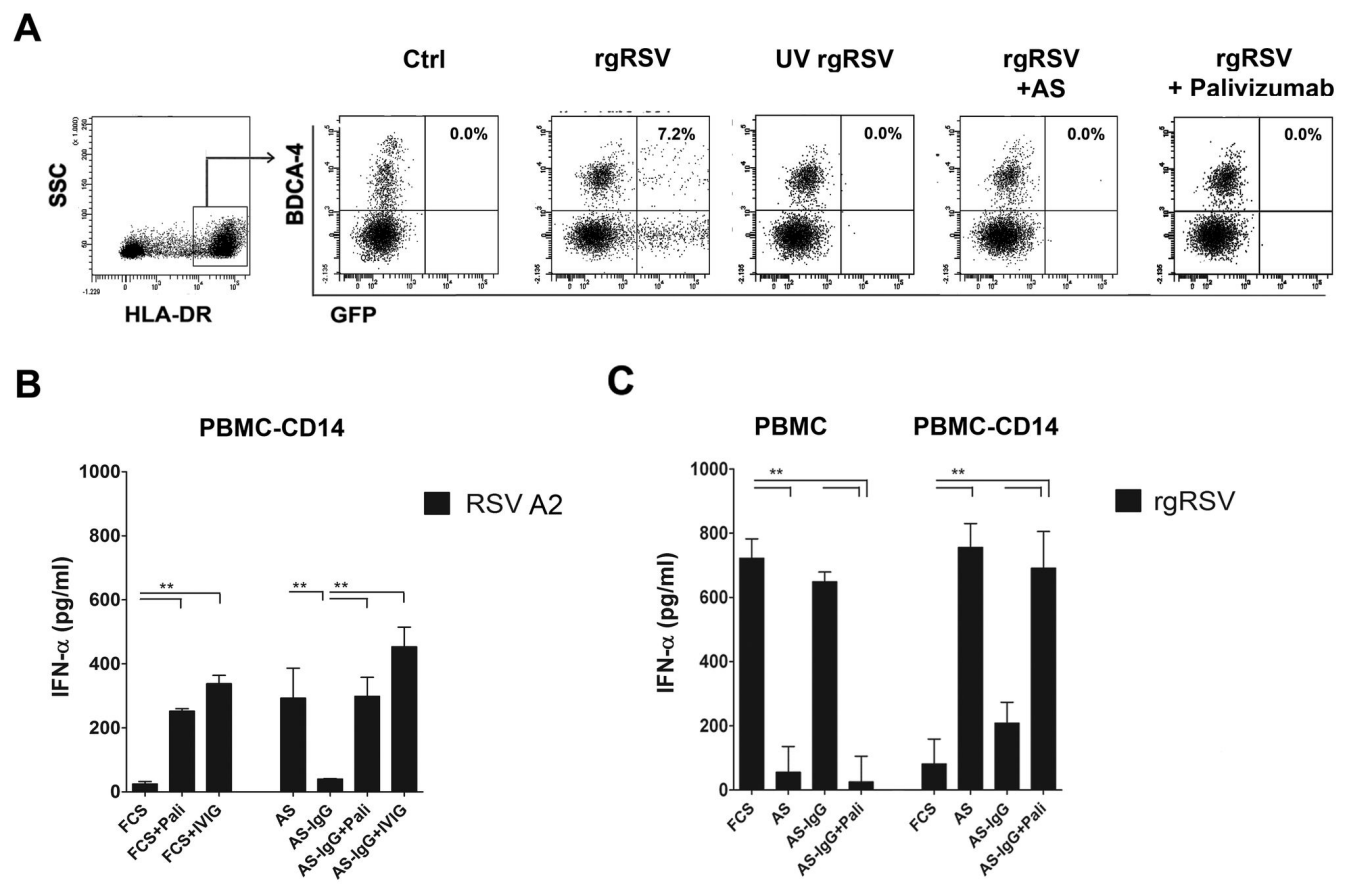
RSV-specific antibodies inhibit RSV-induced IFN-α production in PBMC, but enhance IFN-α production in CD14^+^ cell depleted PBMC. (**A**) Inhibition of RSV infection in pDC. Lineage^neg.^, MHC-II^high^, BDCA-4^+^ pDC are partially infected with rgRSV224 after a period of 20 hours. Infection is blocked after UV inactivation, after neutralization in 10% fresh human serum or in 5µg/ml Palivizumab. Similar results were obtained with sera from all donors used during our studies. IFN-α in supernatant of RSV-A2 exposed PBMC-CD14^+^ cell cultures (**B**) and rgRSV224 exposed PBMC-CD14^+^ cells or PBMC, (**C**) was measured after 20 hrs. The role of virus specific antibodies on the cytokine response was tested by removing IgGs from AS with protein G Sepharose^®^ beads and reconstitution with 2 mg/ml IVIG (**B**) or 5 µg/ml palivizumab (**B**, **C**). Experiments represent the mean ± SEM of experiments performed in 4 different donors and were analyzed using one way ANOVA followed by a Bonferroni post-test, **P <0.01.

We next addressed the question which component in human serum altered the cytokine responses induced by RSV in PBMC in comparison to the responses observed in medium supplemented with FCS. Everyone has been exposed to RSV by three years of age and is re-exposed frequently after. Therefore, adult sera contain RSV-specific neutralizing and non-neutralizing antibodies. In every donor we tested, 10% serum or 5 µg/ml of the RSV-F specific monoclonal Ab palivizumab completely blocked RSV infection in pDC (Figure **5A**), CD14^+^ monocytes and A549 cells (data not shown). Therefore, we evaluated whether RSV-specific antibodies in autologous serum affected IFN-α production in RSV-exposed PBMC and PBMC-CD14^+^ cells. In IgG depletion-reconstitution studies (reconstitution with polyclonal IgG, IVIG), (Figure **5B**) or virus specific monoclonal antibody palivizumab, (Figure **5 B, C**), we confirmed the role for RSV-specific antibodies in the AS dependent IFN-α responses. Interestingly, RSV-specific antibodies inhibited IFN-α production in RSV-exposed PBMC, but enhanced IFN-α production in CD14^+^ cell depleted PBMC (Figure **5C**).

### CD14^+^ monocytes inhibit IFN-α production via the TLR7 route triggered by Ab-RSV complexes in pDC

**Figure 6:**
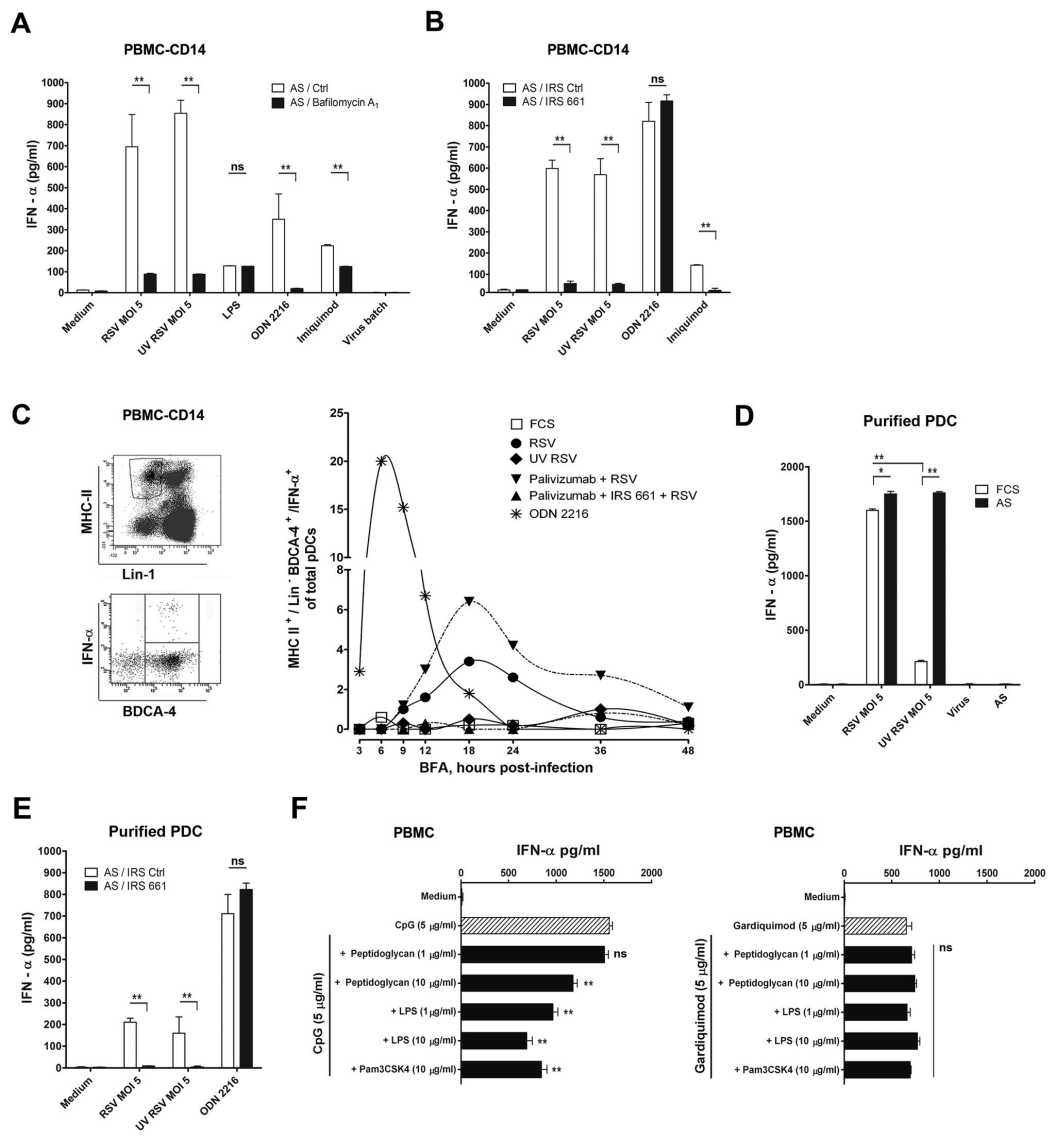
CD14^+^ monocytes inhibit IFN-α production triggered by Ab-RSV via TLR7 in pDC. (**A**) IFN-α production in CD14^+^ cell depleted PBMCs induced by Ab-RSV complexes, TLR9 ligand ODN 2216 and TLR7 ligand imiquimod is decreased by blocking endosomal acidification with 50nM Bafilomycin A_1_. (**B**) Ab-RSV-induced IFN-α production in CD14^+^ cell depleted PBMCs was abrogated in the presence of immune regulatory sequence (IRS) 661 (1.4 µM) a specific blocking agent for endosomal TLR7 and not by a scrambled control nucleotide. (**C**) pDC are the source of Ab-RSV induced, TLR7 mediated production of IFN-α, as shown by intracellular staining for IFN-α in Lineage (CD3^neg.^, CD14^neg.^, CD19^neg.^, CD16^neg.^, CD56^neg.^, Lin-1), MHC-II^high^, BDCA-4^+^ cells. The inhibitor brefeldin A was added at different time points post infection (the time points when BFA was added are given on the X-axis). Cytokines were allowed to accumulate for 10 hrs. after addition of BFA. (**D**) Purified pDC (obtained by negative selection removing CD3^+^, CD19^+^ and CD16^+^ cells from fresh PBMC, followed by FACS purification of the BDCA-4^+^ cell population, which resulted in > 95% pure pDC) produce IFN-α upon infection with RSV. This response is abrogated after UV inactivation of RSV. In AS, both live RSV and UV-inactivated RSV induced IFN-α production to a similar extent. One representative experiment out of two performed with pDC isolated from two different donors is shown. (**E**) IFN-α production by Ab-RSV in purified pDC is blocked by IRS661 (1.4µM). (**F**) TLR1,-2 (PAM3CSK4, Peptidoglycan) and TLR4 (LPS) ligands suppress TLR9-triggered (ODN 2216) IFN-α production, but do not affect TLR7 (Gardiquimod) induced IFN-α production. All data represent mean ± SEM of triplicate measurements within 1 donor and analyzed using one way ANOVA followed by a Bonferroni post-test. ns not significant, *P< 0.05, **P <0.01. Experiments were performed in 3 different donors with similar results.

We next performed experiments to determine the pathway of RSV induced IFN-α production and the dual role of virus specific antibodies in PBMC and CD14^+^ cell depleted PBMC cultures. Single stranded viruses stimulate the production of IFN-α via different mechanisms i.e.; TLR7 recognizes viral RNA delivered to endosomal compartments in pDC and viral infection can elicit IFN-α responses via cytoplasmic sensors. Endosomal presence of double stranded RNA can also trigger type I IFN response via TLR3. Therefore, the mechanism by which a virus interacts with a cell and the route of cell entry will affect the outcome of the innate response induced. From the experiments described in Figure **1B**, **4C** and **5C** it was concluded that in PBMC, live RSV in the absence of virus specific antibodies induced IFN-α, suggesting that an infection mediated process might be involved. In contrast IFN-α production in CD14^+^ cell-depleted PBMC cultures in the presence of serum did not depend on infection because UV-RSV and live RSV induced equal amounts of IFN-α (Figure **4C**). Because pDC depletion aborted the latter IFN-α response (Figure **4C**) we hypothesized that antibody-mediated internalisation and endosomal TLR7 triggering might be the mechanism by which pDC produced IFN-α in PBMC-CD14^+^ cell cultures. To test this hypothesis we blocked endosomal acidification with bafilomycin A_1_, a procedure that abrogates endosomal TLR7 and TLR9 mediated IFN-α production [38]. Indeed when endosomal acidification was abrogated by bafilomycin A_1,_ the Ab-mediated anti-viral IFN-α response was blocked in PBMC-CD14^+^ cells (Figure **6A**). In addition the specific TLR7 inhibitor, IRS661 blocked the IFN-α response induced by RSV immune complexes, while a scrambled control nucleotide did not (Figure **6B**). Furthermore, intracellular staining for IFN-α production identified lineage (CD3,-19,-14,-16,-56)^neg.^, MHC class II^+^, BDCA-4^+^ pDC as the source of IFN-α when palivizumab-RSV complexes were incubated with CD14^+^ cell depleted PBMC (Figure **6C**). A lower amount of IFN-α production induced by live RSV not neutralized by palivizumab was also observed with similar kinetics (Figure **6C**). Both the Ab-mediated and the infectious route of RSV-induced IFN-α occurred with slower kinetics than the production of IFN-α after TLR9 ligation by ODN 2216 (Figure **6C**). The production of IFN-α in response to RSV infection and the non-infectious Ab-mediated route via TLR7 were confirmed in experiments wherein we used purified pDC (Figure **6D**). Also in purified pDC we found that live RSV infection elicited IFN-α production that was abrogated by UV irradiation of RSV. In contrast, in autologous serum that contains RSV specific antibodies, both live and UV-RSV elicited equal amounts of IFN-α, a response that could be specifically abrogated by TLR7 blocking oligonucleotide IRS 661 (Figure **6E**). 

 It is not directly clear why this antibody-mediated route targeting RSV to endosomal TLR7 in pDC is less efficient in PBMC (Figure **4C**, **5C**). One explanation could be a mechanism similar to one that has been described for bacteria, where TLR2 and TLR4 ligands in *E. coli* lysates induced Prostaglandin (PG) E and IL-10 production in monocytes with a synergistic suppressive effect on IFN-α production by bacterial double stranded DNA in PDC [39]. Thus substantial amounts of IFN-α are produced by purified pDC exposed to *E. coli* lysates, but not in PBMC exposed to the same lysate. Because RSV has been reported to trigger a TLR4 response via RSV-F [14,40], we performed several experiments to test whether such a mechanism via TLR4 (and or TLR2) triggers could also decrease the TLR7 induced IFN-α response by RSV. However, we were unable to recover TLR7 mediated antiviral IFN-α response in PBMC, induced by RSV immune complexes, by inhibiting prostaglandin production (via cyclooxygenase inhibitor indomethacin), IL-10 signalling (via Ab-mediated IL-10R blocking) nor by combination of both treatments (data not shown). Also attempts to mimic the viral TLR4, TLR7 dual stimulation with synthetic ligands did not reveal a suppressive effect of TLR1/2 or TLR4 ligands on TLR7 induced IFN-α response, while the TLR9 mediated IFN-α response was significantly supressed by these ligands (Figure **6F**). The suppressive mechanism of CD14^+^ monocytes on IFN-α production by Ab-RSV complexes in pDC remains unresolved. 

### IFN-α production induced by live RSV in PBMC depends on IFNAR signalling

**Figure 7:**
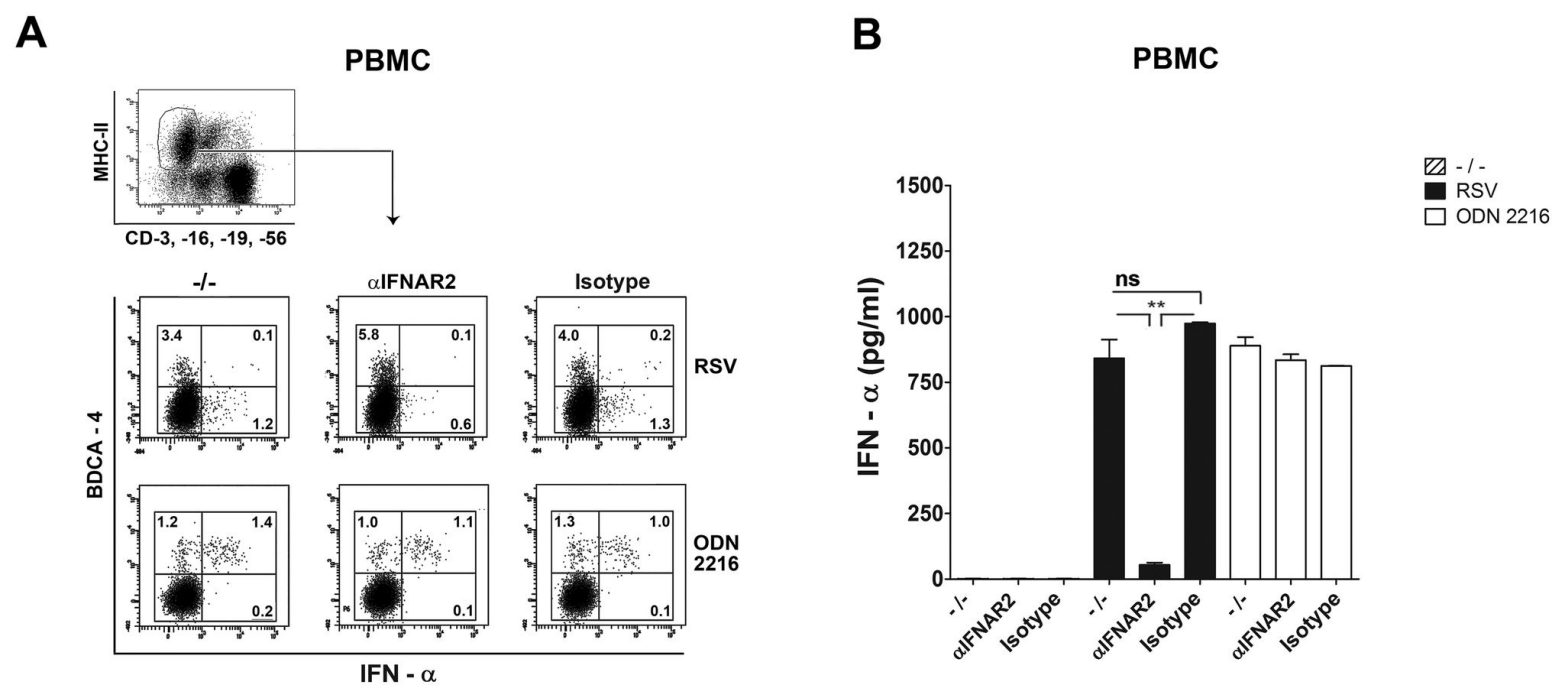
IFN-α production induced by live RSV in PBMC depends on IFNAR signalling. PBMC were exposed to live RSV in the presence of interferon-α/β receptor (IFNAR) blocking antibody (5µg/ml), isotype control Ab or no Ab. Levels of IFN-α were determined via intracellular staining (**A**) or in 20hrs. supernatant by ELISA (**B**). IFN-α was trapped intracellular by BFA treatment initiated 6 hrs. after RSV infection, or at t=0 for the ODN 2216 control stimulus, because of different kinetics of anti-viral and ODN elicited IFN-α response. For both stimuli, intracellular staining for IFN-α was performed in CD3^neg.^, CD16^neg.^, CD19^neg.^, CD56^neg.^, MHCII^+^, BDCA-4^+^ cells after 10 hrs. BFA treatment. Experiments were performed in 3 different donors with similar results. Data represent the mean ± SEM of triplicate measurements within 1 donor and were analyzed using one way ANOVA followed by a Bonferroni post-test. ns not significant, **P <0.01.

After showing that Ab-neutralized RSV is targeted to endo-lysosomal compartments in PDC, where it activates TLR7 mediated IFN-α production, we next addressed the route of IFN-α production by *live* non-neutralized RSV in PBMC. We first attempted to define the cell type involved in IFN-α induction. IFN-α production in PBMC is abrogated when RSV is UV-inactivated, in the presence of neutralizing antibodies, or by depletion of either CD14^+^ monocytes or pDC (Figure **4C**). To explain the role of CD14^+^ cells in the production of the type I interferon by pDC, we reasoned that RSV infection of monocytes may be required for the production of IFN-β, leading to IFN-α production through IFN-α,β receptor (IFNAR) signalling, JAK-STAT activation and an interferon regulatory factor 7 (IRF7) mediated pathway [41,42]. Such a role for monocytes would be in accord with the fact that they are the cell type within PBMC that are most easily infected by RSV (Figure **2**). To test this hypothesis, we performed experiments in which the secondary IFN-α pathway was blocked by an IFNAR blocking antibody. Figures **7A and B** both show that indeed RSV-induced IFN-α production is decreased in the presence of IFNAR blocking Ab at 5µg/ml (a concentration of 1µg/ml blocked the cytokine response by 30%, data not shown), while IFN-α production induced by ODN 2216 (IFN-α production via endo-lysosomal TLR9 activation is independent of the indirect IFNAR mediated route) was not affected by blocking the autocrine or paracrine type I IFN amplification loop. Furthermore, ODN 2216 clearly induced IFN-α production in BDCA-4^+^ pDC, whereas in RSV infected PBMC cultures, low numbers of BDCA-4^+^ cells as well as BDCA-4^neg.^ cells contributed to the IFN-α response. However, in the experiments shown in Figure **7A**, the intracellular cytokine staining for IFN-α was performed within the first 6 hrs. of the cultures of PBMC exposed to ODN 2216, whereas RSV-induced IFN-α was measured by starting the Brefeldin A treatment to accumulate cytokines from 12 hrs. post infection. This was done because the kinetics of the response differs for these treatments (Figure **6C**). When Brefeldin-A treatment was postponed in the ODN 2216 treated PBMC cultures, low numbers of IFN-α positive cells were found that were all BDCA-4 negative. These results indicate that BDCA-4 might be down regulated in time during culture and might not be a stable marker to identify pDC after prolonged culture in the context of PBMC with TLR ligands or ligands for other PRRs. For this reason we were unable to exclude pDC nor confirm pDC as the responding population. Also CD14 is down-regulated on monocytes in PBMC cultures and strongest in RSV infected samples. Therefore, CD14 staining could also not be used to determine the role of monocytes as a possible source of IFN-α. In conclusion, RSV infection elicits IFN-α production in pDC via an IFNAR dependent mechanism in the context of PBMC. The involvement of monocytes in this process is suggested by the fact that CD14^+^ cell depletion abrogates the IFN-α production (Figure **4C** and **5C**). Because UV-inactivation abrogates the IFN-α response in PBMC, combined with the fact that monocytes are the cells that are most efficiently infected (Figure **2**), this suggests the involvement of an infection mediated route, resulting in an early source of type I interferon production by monocytes that triggers IFN-α production by IFNAR binding on pDC.

### CD14 plays a crucial role in the IFN-α production induced by RSV in PBMC

**Figure 8:**
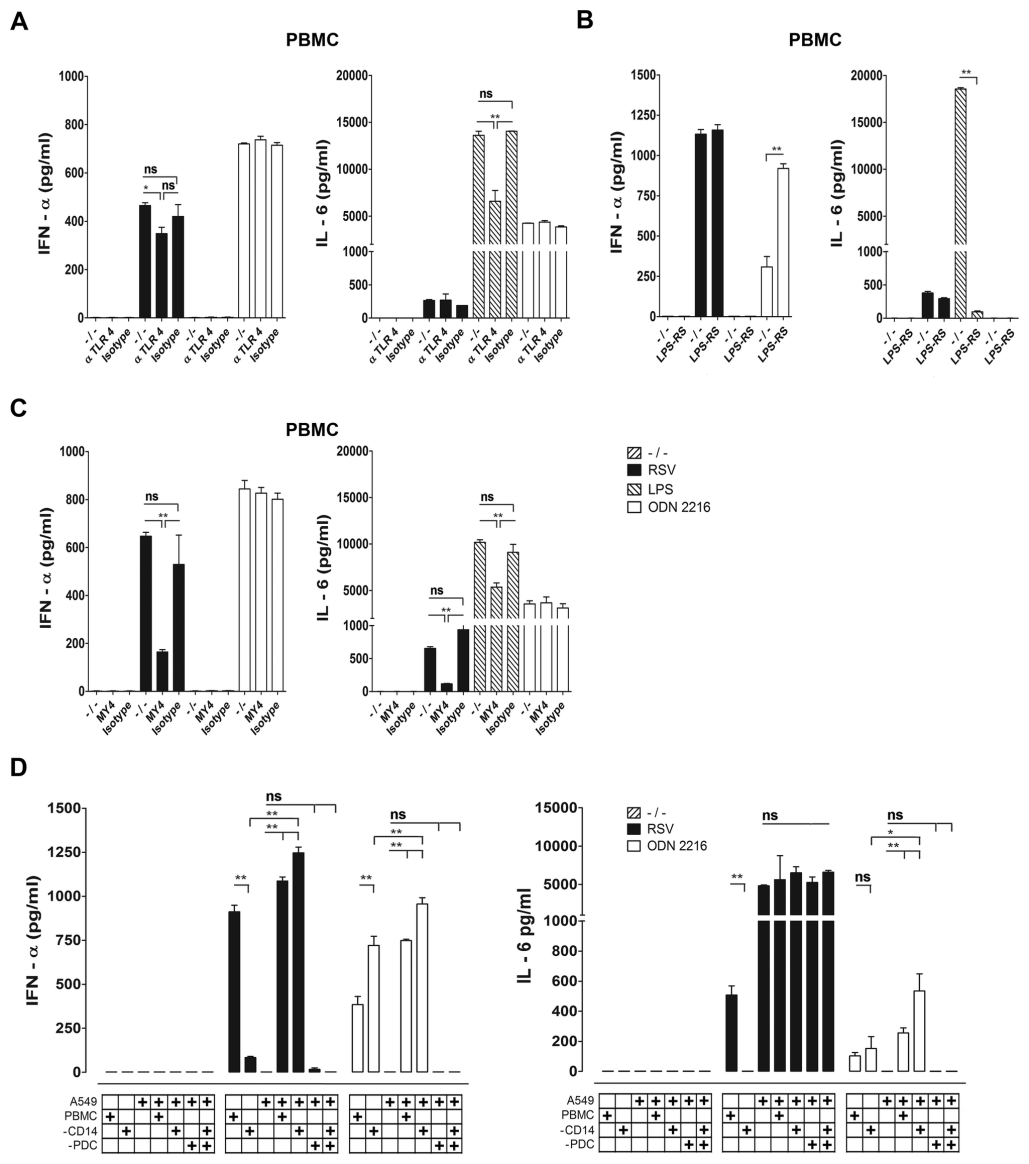
IFN-α production induced by live RSV in pDC depends on CD14. The role of the TLR4/CD14/MD2 complex in the IFN-α and IL-6 response by PBMC after RSV infection (strain A2, MOI 5) was investigated (**A**) with a blocking monoclonal antibody specific for human TLR4 (20µg/ml), (**B**) the MD2 antagonist lipopolysaccharide from the bacterium *Rhodobacter* *sphaeroides* (1µg/ml) or (**C**) via neutralization of CD14 with monoclonal antibody MY4 (10µg/ml). (**A**-**C**) Cytokine responses were measured by ELISA. Control stimuli; LPS: 10ng/ml, ODN: 5µg/ml. (**D**) A549 epithelial cells were co-cultured with PBMC, CD14^+^ cell depleted PBMC, pDC depleted PBMC, or CD14^+^ and BDCA-4^+^ double depleted PBMC, in the presence of RSV at MOI 5, ODN 2216 or no stimulus, for a period of 20 hrs. IL-6 and IFN-α production were measured by ELISA. Experiments were performed in 3 different donors with similar results. Data represent the mean ± SEM of triplicate measurements within 1 donor and were analyzed using one way ANOVA followed by a Bonferroni post-test. ns not significant, *P<0.05, **P<0.01.

The observation that UV-irradiation and virus neutralization by palivizumab prevented IFN-α production by pDC exposed to RSV in PBMC (Figures **5C** and **6C**), could suggest that infection with RSV might be required for IFN-α production. However, there may be another possible explanation for the abrogated IFN-α production when RSV is inactivated by UV irradiation or neutralized by antibodies. It has been reported that RSV-F interacts with the TLR4 complex, which could result in IFN-β production and has been shown to induce TNF-α and IL-6 production in human monocytes [14,40]. It is possible that F specific antibodies or UV treatment (by affecting the structure of the labile F molecule) could block TLR4 signalling. Therefore, Ab-mediated neutralization or UV-inactivation did not unequivocally distinguish the surface TLR-mediated or infection related innate immune pathways. To determine whether RSV-F/TLR4 interaction contributed to cytokine production in our experiments, we measured cytokine responses induced by RSV in PBMC cultures in the presence or absence of CD14-, or TLR4-blocking Abs and a LPS antagonist, LPS-RS, from *Rhodobacter sphaeroides*, that inhibits LPS and RSV-F interaction with TLR complex cofactor MD2 [40,43]. Both antibodies and LPS-RS blocked the LPS induced TLR4-mediated IL-6 response in PBMC (Figure **8A, B and C**), while only MY4, the CD14 blocking antibody, decreased IL-6 and IFN-α production in PBMC exposed to RSV (Figure **8C**). CD14-neutralizing antibody slightly decreased the level of infection of monocytes by rgRSV224 (data not shown). However, it seems unlikely that the marginal effect on the RSV infection of the monocyte population explained the significant inhibition of IFN-α production by PBMC. Therefore, our data are consistent with a cell surface TLR-complex mediated role during early IFN-type 1 production by RSV, although direct viral interaction with TLR4 or MD2 might not occur. Because A549 cells, that do not respond to inactivated RSV via TLR4, did produce substantial amounts of IFN-β upon RSV infection (Figure **1**), we tested whether RSV-infected A549 cells could restore IFN-α production in monocyte depleted PBMC. In Figure **8D** we show that indeed the IFN-α responses can be restored by co-culture of CD14^+^ cell depleted PBMC with A549 exposed to live RSV and that pDC are the source of this IFN-α.

## Discussion

**Figure 9:**
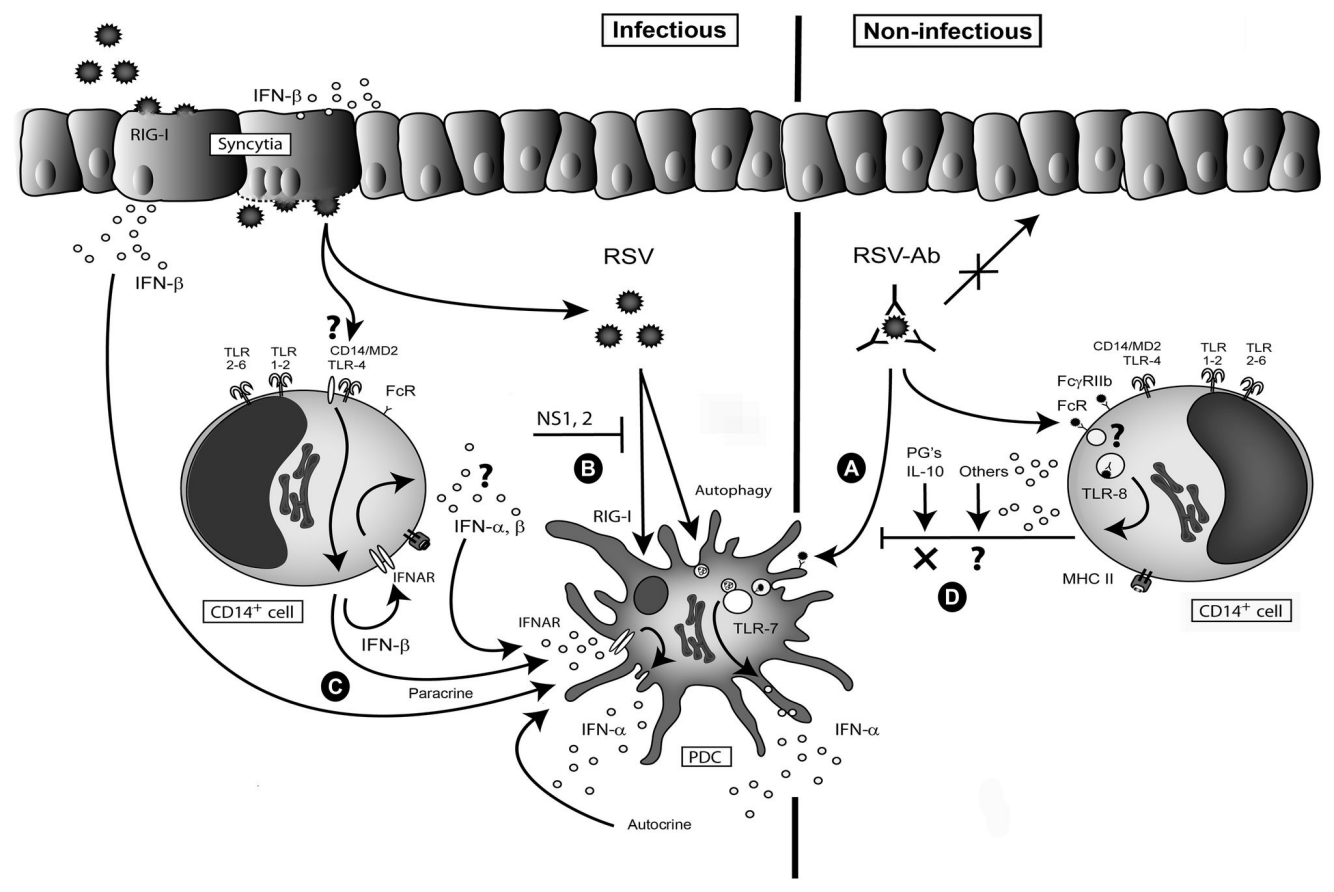
Cellular cross talk and the role of antibodies during IFN-α production. pDC produce IFN-α via multiple pathways. i: TLR7 activation after Ab-mediated uptake of RSV (**A**), ii: via infection with RSV in purified pDC (**B**) or iii: indirect via IFNAR-mediated signalling triggered by IFN-β produced by RSV-infected A549 (as a model for airway epithelium) or RSV-exposed CD14^+^ cells (**C**). In the context of PBMC the presence of CD14^+^ monocytes suppresses the production of IFN-α by pDC upon exposure to Ab-RSV complexes. (**D**). The mechanism of the suppression by monocytes of this TLR7-mediated response elicited by Ab-RSV complexes in pDC remains unresolved, but appears not to be mediated via PG’s and IL-10, mediators that suppress TLR9 induced IFN-α production when multiple PAMPS in bacterial lysates simultaneously trigger TLR9 and TLR2/4 responses in PBMC.

In the present work we showed that RSV-specific (neutralizing) antibodies play a crucial role in dictating the nature of innate immune reactions to RSV. Moreover, the innate immune reaction elicited by RSV depends on cell specific modes of interaction with RSV. These different modes of interaction and the specific access this creates towards pattern recognition receptors present in a particular cell type, lead to different innate immune pathways. As reported earlier, epithelial A549 cells produced mainly inflammatory cytokines (IL-6, TNF-α, IFN-β) in a process that depended on RSV infection and is presumably mediated via RIG-I [47,48]. pDC produced type I interferon (IFN-α) via TLR7 activation after Ab-mediated uptake of RSV (Figure **9A**), or via infection with RSV in purified pDC (Figure **9B**). CD14^+^ cells were the main producers of inflammatory cytokines in PBMC.

We further show that the inflammatory response induced by RSV depends on the cellular context during virus exposure and immune status, i.e. the presence or absence of virus specific (neutralizing) antibodies. This was demonstrated by the role of CD14^+^ cells in suppressing IFN-α production by pDC upon Ab-RSV exposure (Figure **9A, D**) and in the absence of antibodies, the indirect mechanism initiated by RSV-infected epithelial cells or RSV-exposed CD14^+^ cells that activated IFN-α release via IFNAR-signalling in pDC (Figure **9C**).

During natural exposure to RSV, airway epithelial cells, alveolar macrophages in the airway lumen and dendritic cells underneath the epithelium are the primary cells initially exposed to RSV rather than PBMC. Moreover, as shown in mouse models, the early innate response causes the influx of DC types into the lung tissue and migration of DC to the draining lymphnodes, followed by T cell activation in the lymphnodes and trafficking to the infected tissue [44]. Clearly, the structural restraints, cell density and trafficking, the sequence of subsequent cellular exposure to RSV and cellular cross-talk in different environments (airway, lung-parenchyma and lymphnode) will affect the outcome of the innate immune response *in vivo*. Indeed *in vivo* mouse models for RSV implicated the contribution of different cell types and PRR systems in murine innate immune responses to RSV. IPS-1 signalling and presumably infection related routes of innate immune triggering, in both immune and non-immune cells, were important in viral clearance. On the otherhand, TLRs (3 and 7) were less important in virus clearance but affected disease parameters like mucus production, presumably via skewing of Th responses towards Th2 or Th17 respectively [17,18,31,45,46].

The *in vitro* experiments described in this study are a simplified model of the cross talk that is likely to occur *in vivo*. In addition, during vaccination the formulation of the vaccine; i.e. method of virus inactivation, omission of certain viral components and/or added adjuvants and different location of entry distant from the airways will complicate the outcome even more. Our simple *in vitro* experimental setup does not allow to make predictions of the exact inflammatory response *in vivo*, where different cell types and cell dynamics may contribute to the response. However, it does demonstrate that *in vitro* analysis using single cell types to predict the potency and safety of attenuated RSV vaccine candidates is a poor approach to test such vaccine candidates.

 We showed a crucial role of CD14^+^ cells in inflammatory cytokine and type I interferon production upon RSV exposure in PBMC. The mechanism by which CD14^+^ cells are triggered to produce cytokines is still controversial. Kurt-Jones et al. first reported the role of RSV-F as a ligand for the TLR4/CD14 complex and the same group later reported that RSV could also trigger TLR2 and TLR6 mediated responses [13,14]. Recently Rallabhandi et al. demonstrated that purified RSV-F interacted with MD2 [40]). However, other groups were unable to confirm TLR4-mediated RSV interactions [49], or were unable to confirm a role of the viral F protein in the process [50]. Importantly, Rallabhandi and colleagues did not show MD2 dependent TLR4 activation with intact RSV. Also in studies *in vivo* using TLR4-deficient mice conflicting results for a role of TLR4 in RSV specific immunity were reported [19,51,52]. It is difficult to reconcile why the results in different laboratories are so different. However, many variables in experimental setup may contribute to different results; i.e. virus culture in HEp versus Vero cells, the use of human monocytes, murine macrophages or reporter cell lines, differences in purification protocols for RSV or RSV-F. TLRs in different species although quite homologous still differ substantially in amino acid residues, changes that lead to differences in leucine-rich repeat organisation and also ligand specificity [53,54]. Moreover, in TLR-mutant or knockout mice it is conceivable that altered lung development [55,56] or differences in microbiota [57] could affect *in vivo* disease development after RSV infection. Therefore, altered immune responses elicited upon viral infection in knockout mice could occur via different mechanisms and do not unequivocally prove (the lack of) interaction of the virus with the TLR studied, as the reason for the altered immune response. Similar caution is warranted in the interpretation of human TLR polymorphisms and their involvement in severity of RSV disease. 

In this study we found that live RSV elicited IL-6, IL-10, IL-8 and TNF-α production in PBMC and not in PBMC-CD14^+^ cells. In contrast to responses in A549 cells UV-RSV still stimulated production of these cytokines although in most experiments in slightly lower amounts. TLR4 blocking antibody in a dose that diminished LPS induced IL-6 production by > 50% did not significantly inhibit IL-6 and IFN-α production by RSV in PBMC, neither did LPS-RS affect RSV-induced IL-6 and IFN-α production, while it did inhibit IL-6 production triggered by LPS completely (Figure **8**). The latter observation suggests that in the context of RSV particles, MD2-RSV-F interaction might not occur in a similar fashion as was reported for purified F protein [40]. We did reproduce the reported effect of blocking CD14 on RSV induced cytokine responses in CD14^+^ cells [14] and show that in addition to IL-6 production also the primary burst of type I interferon in PBMC cultures depends on CD14. CD14 is an important cofactor for the activation of endosomal TLRs. In addition to be required for the TRIF/TRAM pathway downstream of TLR4 [58], CD14 has been reported to associate with TLR7, TLR8 and TLR9 and to act as a co-receptor for these TLRs in endosomes [59]. Because CD14 is dispensable for cytoplasmic sensing of viral infection, our data suggest that IFN-α production by pDC in the context of PBMC depends on a TLR mediated route. It remains to be established which of the TLR pathways is involved.

pDC and type I interferons exert a crucial role in regulating adaptive immunity [60–63] and have been implicated in virus clearance and protection against immune mediated pathology caused by RSV infection [64]. It is still unclear why IFN-α induction is ineffective in PBMC-CD14^+^ cells a cell mixture wherein pDC are present. We confirmed earlier studies that have shown that purified pDC could produce IFN-α when exposed to infectious RSV (Figure **6D**), although the amount of IFN-α might be suppressed once viral non-structural proteins are expressed in infected pDC [26,27,35,65]. In contrast to influenza virus [66] and vesicular stomatitis virus [67], RSV infection and propagation does not follow an endo-lysosomal route [68]. The absence of endosomal uptake as the natural route of cell entry might explain the absence of TLR7-mediated IFN-α production by UV-inactivated RSV. Ab-mediated uptake presumably enhances access of UV-RSV and live RSV to endosomal TLR7 in pDC explaining the increased IFN-α production when virus specific antibodies are present (Figure **9A**). Moreover, the neutralizing effect of RSV-specific monoclonal Ab palivizumab or polyclonal serum antibodies prevents infection and expression of viral non-structural proteins that interfere with type I interferon production pathways [26,35]. However, we observed that live RSV can elicit IFN-α production in purified pDC in an IRS661 sensitive fashion (Figure **6E**). Most likely this IFN-α production might be the result of autophagy, [69], that occurs after infection of low numbers of pDC which is then followed by a second burst of IFN-α via IFNAR signalling in non/infected bystanders (Figure **9B**). This process has been described before and has been shown to critically depend on the density of *in vitro* cultured pDC [70]. It is possible that in the context of PBMC-CD14 the pDC are too low in numbers for this process to occur.

 By means of early expressed nonstructural proteins, RSV evades the type I interferon pathway and antiviral response in infected cells by suppression of the activation and nuclear translocation of interferon regulatory factor 3 [35,71]. However IRF3 activation occurs in the first 10 hrs. of infection before substantial amounts of NS proteins are expressed. This window of IRF3 activation suffices to contribute together with NF-κB activation to the production of IFN-β in A549 and presumably CD14^+^ cells which in turn potentially stimulates secondary IFN-α responses via IFNAR signaling in pDC (Figure **9C**). In addition NS2 suppresses Stat2 levels interfering with IFN-α/β signaling via IFNAR, the secondary route, leading to expression of interferon stimulated genes and a secondary burst of type I interferons [72]. Therefore, uninfected bystander pDC are an important source of type I interferon production. 

In summary, our findings show that the interaction of RSV with a mixture of primary human cells (PBMC) leads to a cytokine response pattern that depends on cellular cross-talk and the presence or absence of RSV specific antibodies. We show a key role for CD14 monocytes in the control of antiviral type I IFN responses to RSV via a direct antibody mediated and an indirect, infection mediated, mechanism. Because RSV specific antibodies are transmitted from mother to child during pregnancy and via breast milk the role of these antibodies should be carefully evaluated with respect to their effect on innate immune responses when new vaccine candidates are tested.

## Materials and Methods

### Ethics Statement

 Human peripheral blood mononuclear cells (PBMCs) were isolated from whole blood donated by healthy adult volunteers. All studies using primary cells were performed after written approval from the ethics Committee of the Medical Faculty of the Utrecht University and after obtaining written informed consent from the donors. 

### Human hematopoietic cell isolation and single cell depletion

*In vitro* experiments were performed using PBMC isolated from EDTA-anticoagulated whole blood isolated by Ficoll-Hypaque density gradient centrifugation (Biochrom, Germany). Specific cell type depletion was achieved by positive selection using antibody labeled magnetic micro beads (from Miltenyi Biotec unless otherwise stated) against CD14 (IgG2a, 130-050-201) and BDCA-4 (IgG1, 130-092-402) following standard procedures. PBMCs were incubated with bead labeled antibody at 4°C for 30 min in deoxygenated PBS with 0.5% BSA and 2 mM EDTA (MACS buffer) and passed through a magnetic separation column once (LD column, 130-042-901; Miltenyi Biotec). Depletion of the cell type of interest and specificity of depletion was confirmed using flowcytometry. Efficiency of depletion was at least 98%. pDC purification by positive selection with BDCA-4 coupled magnetic beads suppressed IFN-α production in our experiments. Therefore, we purified pDC by negative selection (by magnetic beads), removing CD3^+^-, CD19^+^- and CD16^+^ cells from freshly isolated PBMC, followed by FACS purification of the BDCA-4^+^ cell population (FACSAria; Becton Dickinson). Purity of isolated pDC was >95%. Cell viability was assessed by trypan blue exclusion. 5x10^4^-10^5^ cells were used per stimulus in duplicate.

### Cell lines and virus

HEp-2 cells were cultured in IMDM (21980-065, Life Technologies, Rockville, MD) and the A549 cell line was cultured in DMEM (31965-080, Life Technologies, Rockville, MD) both supplemented with 2 mM l-glutamine, 25 mM HEPES buffer, 5% FCS, and 1% penicillin/streptomycin. RSV strain A2 (VR-1540, ATCC), RSV Long strain (VR-26, ATCC), RSV patient strain (A, 13N01), RSV patient strain (A, 16N01) and recombinant GFP expressing RSV A2 (rgRSV224, GFP gene inserted into a naturally occurring BstXI site before the gene coding for non-structural protein 1 (NS1), kindly provided by Dr. M.E. Peeples and manufactured as previously described by Hallak et al.) [37], were grown on HEp-2 cells (CCL-23, ATCC) in 1% FCS, purified by polyethylene glycol 6000 precipitation, resolved in phosphate-buffered saline (PBS) in the presence of 10% sucrose and stored in liquid nitrogen. The 50% tissue culture–infective dose (TCID-50) was determined post-titration in HEp-2 cells. 

### RSV binding and infection

For cell culture experiments, freshly isolated, specific cell depleted or whole PBMC suspensions (4×10^5^) were cultured in round-bottom 96-wells plates (Costar) in a final volume of 200µl. For RSV binding experiments, cells were incubated for a period of 1hr at 4°C with rgRSV224 in IMDM supplemented with, 5% FCS, 2 mM L-glutamine, 25 mM Hepes buffer and 1% penicillin/streptomycin. RSV infection studies were performed with live or UV-inactivated RSV (2300 µW/cm^2^ of 254 nm UVA light for 10 min.) and measured after an overnight (20-24h) incubation period at 37°C in 5% CO_2_, or as indicated in the Figure legends. Supernatants were collected and stored at −20°C until further analysis.

### Cell identification and cytokine production

To identify different cell populations, cultured single cell suspensions were washed with PBS containing 2% FCS, 2mM EDTA and 0.02%NaN3 (FACS buffer), blocked with 10% human AB serum for 20 min. at 4°C to reduce non-specific binding and stained for 4- or 5-color flow cytometry with the following antibodies (from BD Biosciences unless otherwise stated) anti-CD3 (clone SK7), anti-CD4 (clone SK3), anti-CD8 (clone SK1), anti-CD11b (clone D12,), anti-CD11c (clone B-ly6), anti-CD14 (clone MϕP9), anti-CD16 (clone 3G8), anti-CD19 (clone 4G7), anti-CD56 (clone NCAM16.2), anti-CD123 (clone 9F5), anti-BDCA-1 (clone AD5-8E7, Miltenyi Biotec), anti-BDCA-4 (clone AD5-17F6, Miltenyi Biotec) or anti-MHC-II (clone G46-6). To identify cell specific cytokine production, cells were first surface stained with specific antibodies to phenotype cell populations before fixation and permeabilisation with CytoFix/CytoPerm (554722, BD) solution and Perm/Wash buffer (554723, BD). Intracellular cytokines were detected with anti-IFN-α (clone 7N4-1) antibody. Stained samples were measured on a FACS Canto II flow cytometer (BD Biosciences, San Diego, CA) and analysed using FACS Diva software (BD, San Diego, CA). 

For detection of cytokines in supernatant, culture supernatants were collected after a 20hrs incubation period, or as indicated in Figure legends. Enzyme-linked immunosorbent assay (ELISA) was used to quantify human IFN-α (IFN-α, -α2a, 2b and 2c, BMS216MST, Bender MedSystems), IFN-β (*Verikine* ^TM^, PBL, 41410-1A), IL-6 (M9316, Sanquin Pelipair), IL-8 (M9318, Sanquin Pelipair) and TNF-α (M9323, Sanquin Pelipair) production in the supernatants. For some experiments a multiplex immunoassay (MIA) of IL-1β, -6, -8, -10, -12, IP-10 and TNF-α in culture supernatant was performed using the Bio-Plex System in combination with Bio-Plex Manager software V.4.1 using five parametric curve fitting (Bio-Rad laboratories, Hercules, California, USA) [73]. 

### Cell stimulation

In experiments in which the effect of autologous serum or RSV specific AB was tested, RSV or control ligands were preincubated for 15 min. at 37°C with 10% autologous serum, 2mg/ml IVIG^®^ or 5ug/ml Palivizumab before co-culture with single cell suspensions. When indicated, IgGs were depleted from serum with Protein G Sepharose beads (P3296, Sigma-Aldrich). The following control ligands (from Invivogen unless otherwise stated) were used; TLR1,-2 (PAM3CSK4, tlrl-pms), TLR2 (Peptidoglycan, tlrl-pgn), TLR3 (Poly I:C, tlrl-pic), TLR4 (ultra-pure LPS, *E. coli* K12), TLR7 (Imiquimod, tlrl-imqs or Gardiquimod, tlrl-gdqs), TLR8 (CL-075, tlrl-c75) and TLR9 (ODN 2216, tlrl-2216). PBMC or A549 cells were co-cultured with RSV for 20 hrs. or different duration as stated in the figure legends.

### Inhibition assays

To determine the involvement of specific innate immune pathways we employed inhibitors to block endosomal acidification (Bafilomycin A_1_, 50nM, Calbiochem) and protein transport (Brefeldin A, 40nM, Sigma-Aldrich). For investigation of TLR4 signalling, a blocking monoclonal antibody specific for human TLR4 (20µg/ml, anti-hTLR4-IgG1, Invivogen), the MD2 antagonist lipopolysaccharide from the bacterium *Rhodobacter sphaeroides* (1µg/ml LPS-RS, Invivogen) or CD14 blocking monoclonal antibody MY4 (10µg/ml, A83482, Beckman Coulter) were used. The human interferon-α/β receptor (IFNAR) was neutralized with a mouse monoclonal antibody (5µg/ml, clone MMHAR-2, PBL Interferon Source). All antibody based neutralizing experiments were performed with matching isotype controls. Endosomal Toll like receptor 7 signalling was blocked with specific immune-regulatory DNA sequences (IRS) to TLR7 (1.4 µM, IRS661: 5′-TGCTTGCAAGCTTGCAAGCA-3′, Eurofins MWG Operon). As a control, a non-TLR-specific oligodeoxyribonucleotide sequence was used in the same concentration as the inhibitor. Cell populations were preincubated with specific (TLR) inhibitors for a period of at least 30 min. at 37°C before adding virus or control TLR ligands.

### Statistical analysis

For all experiments, data are expressed as the mean **^+^**/- standard error of the mean (SEM). Measurements were compared using a one-way or two-way analysis of variance (ANOVA) followed by Bonferroni’s post-hoc analysis. Non parametric analysis was performed using the Kruskal-Wallis test followed by Dunn's Multiple Comparison Test. A P-value of <0.05 was considered to be statistically significant.

## Supporting Information

Figure S1 **Cytokine responses in PBMC exposed to RSV A 13NO1 and 16NO1 at MOI 1.** PBMC cultured with the RSV strains 13N01 and 16N01 at MOI 1, UV inactivated RSV, or RSV neutralized in autologous serum. After 20 hrs. incubation, cytokines were measured in supernatant by ELISA. Experiments were performed in 3 different donors with similar results. Data shown represent the mean ± SEM of 3 measurements within 1 representative donor and were analyzed using one way ANOVA followed by a Bonferroni post-test, *P< 0.05, **P <0.01.
(TIF)
