# Supplementary figures and images for: Respiratory Syncytial Virus Induced Type I IFN Production by pDC Is Regulated by RSV-Infected Airway Epithelial Cells, RSV-Exposed Monocytes and Virus Specific Antibodies
Source: PLoS One. 2013 Nov 26;8(11):e81695. doi: 10.1371/journal.pone.0081695 (PMC3841124; doi:10.1371/journal.pone.0081695)

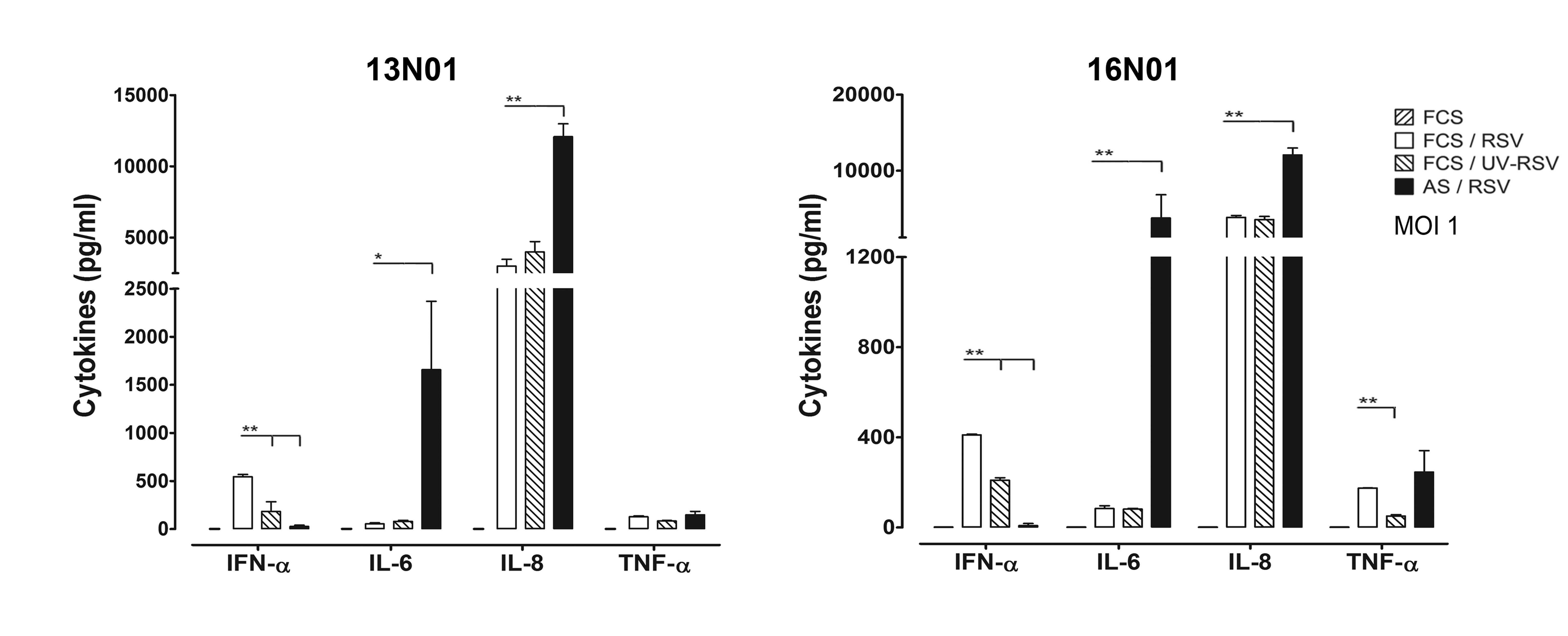

Supplement: Figure S1 — Cytokine responses in PBMC exposed to RSV A 13NO1 and 16NO1 at MOI 1. PBMC cultured with the RSV strains 13N01 and 16N01 at MOI 1, UV inactivated RSV, or RSV neutralized in autologous serum. After 20 hrs. incubation, cytokines were measured in supernatant by ELISA. Experiments were performed in 3 different donors with similar results. Data shown represent the mean ± SEM of 3 measurements within 1 representative donor and were analyzed using one way ANOVA followed by a Bonferroni post-test, *P< 0.05, **P <0.01. (TIF) [file pone.0081695.s001.tif]
